# Long-term CMV monitoring and chronic rejection in renal transplant recipients

**DOI:** 10.3389/fcimb.2023.1190794

**Published:** 2023-06-13

**Authors:** Shoko Ishikawa, Masayuki Tasaki, Kazuhide Saito, Yuki Nakagawa, Masahiro Ikeda, Kota Takahashi, Yoshihiko Tomita

**Affiliations:** ^1^ Division of Urology, Department of Regenerative & Transplant Medicine, Graduate School of Medical and Dental Sciences, Niigata University, Niigata, Japan; ^2^ Department of Urology, Juntendo University Graduate School of Medicine, Tokyo, Japan; ^3^ Takahashi Memorial Medical Institute, Tokyo, Japan

**Keywords:** kidney transplantation, chronic rejection, CMV, monitoring, long-term

## Abstract

**Introduction:**

Cytomegalovirus (CMV) is well established to be an independent risk factor for graft loss after kidney transplantation (KTx). Monitoring for CMV in the chronic phase is not defined in the current guideline. The effects of CMV infection, including asymptomatic CMV viremia, in the chronic phase are unclear.

**Methods:**

We performed a single-center retrospective study to investigate incidence of CMV infection in the chronic phase, defined as more than 1 year after KTx. We included 205 patients who received KTx between April 2004 and December 2017. The CMV pp65 antigenemia assays to detect CMV viremia were continuously performed every 1–3 months.

**Results:**

The median duration of the follow-up was 80.6 (13.1–172.1) months. Asymptomatic CMV infection and CMV disease were observed in 30.7% and 2.9% in the chronic phase, respectively. We found that 10–20% of patients had CMV infections in each year after KTx which did not change over 10 years. The history of CMV infection in the early phase (within 1 year after KTx) and chronic rejection were significantly associated with CMV viremia in the chronic phase. CMV viremia in the chronic phase was significantly associated with graft loss.

**Discussion:**

This is the first study to examine the incidence of CMV viremia for 10 years post KTx. Preventing latent CMV infection may decrease chronic rejection and graft loss after KTx.

## GRAPHICAL ABSTRACT

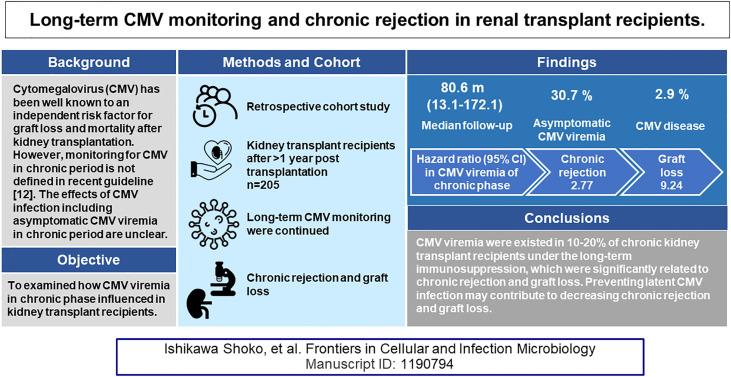

## 1 Introduction

Cytomegalovirus (CMV) has been well known as an independent risk factor for graft loss and mortality after kidney transplantation (KTx). The risk of CMV infection is related to several factors (Tang et al., 2022), including intensity of immunodeficiency (Manuel et al., 2013; Meesing and Razonable, 2018), CMV serology matching between the donor (D) and recipient (R) (Eid and Razonable, 2010), immunosuppressive drugs (Lui and Halloran, 1996; Razonable et al., 2001), and CMV universal prophylaxis or preemptive therapy. The indirect effect of CMV infection on transplant outcome are known, such as graft rejection, opportunistic infection, and even death of transplant recipients. However, the majority of clinical analyses were focused on CMV infections in the early phase after KTx because of the occurrence frequency. Even though the late-onset CMV infection is an important issue after KTx, the data about the CMV infection in the chronic phase are lacking. Previous reports regarding late-onset CMV infection and CMV viremia had short observation periods (< 2.0 years after KTx in most studies), and the details of CMV monitoring were missing or limited (Viot et al., 2015; Selvey et al., 2017; Rahimishahmirzadi et al., 2021; Chaudhari et al., 2022). A previous study retrospectively analyzed the long-term impact of CMV infection over a period of 10 years after KTx (Browne et al., 2010), however, CMV monitoring was performed only when the patients had signs or symptoms, which was the same selection condition as in other reports. The effects of CMV infection, including asymptomatic CMV viremia, in the chronic phase are unclear. Monitoring for CMV in the chronic phase is not prescribed in a recent guideline (Razonable and Humar, 2019). In our institute, we have routinely and frequently monitored CMV infection in the chronic phase after KTx. In the present study, we examined how CMV viremia in the chronic phase influenced kidney transplant recipients.

## 2 Materials and methods

### 2.1 Patients

This was a single-center retrospective study that examined the presence and consequences of CMV infection in the chronic phase, defined as more than 1 year after KTx. We included 313 patients who received KTx between April 2004 and December 2017. We excluded 102 patients whom we could not continuously follow up after KTx. Of the remaining 211 patients, six cases were excluded because CMV-antigenemia (Ag) was not examined in them in the chronic phase. Thus, we finally enrolled a total of 205 patients. This study was approved by the Ethics Committee of the Faculty of Medicine of the Niigata University (registration number: 2018-0287). Clinical and laboratory information was extracted from electronic databases and patients’ medical records.

### 2.2 CMV antigenemia assay and definition of CMV infection

We used the CMV pp65 (C10/C11) Ag assay to detect CMV viremia. CMV Ag assay was performed on-site laboratory and all samples were processed within few hours in this study. In this assay, CMV positive cells are counted in two slides that contain 150,000 leukocytes each. In this study, we defined positive CMV-Ag was diagnosed by the presence of at least one CMV-positive cell per 150,000 leukocytes in the assay. CMV-Ag was routinely monitored weekly for 2–3 months after KTx and then monthly, for up to 1 year. Thereafter, the frequency of CMV monitoring was every 1–3 months in the chronic phase with some exceptions.

We analyzed the degree of CMV-Ag, number of measurements of CMV-Ag, clinical signs caused by CMV infection, and frequency of treatment for CMV in the chronic phase.

In our institution, we gave prophylactic treatment for CMV after KTx as follows: intravenous ganciclovir (GCV) and γ-globulin in the perioperative phase followed by oral valganciclovir (VGCV) for CMV seronegative recipients or low dose oral VGCV for CMV seropositive recipients for at least 6 months. Before 2016, we used acyclovir (ACV) as a substitute of GCV (because the Japanese health insurance system started to cover VGCV as CMV prophylaxis in KTx recipients only in 2016). VGCV and ACV dosing regimens were 450 mg qd and 200 mg bid, respectively, from the start of treatment, and these were adjusted depending on the renal function and adverse reactions.

In the chronic phase, we treated patients if they suffered from CMV diseases or if CMV-Ag values increased. We treated CMV infection in the chronic phase either by re-starting ACV, VGCV, or intravenous GCV (in case of severe disease), or by increasing the dose of VGCV in patients who had continued prophylactic treatment even in the chronic phase. CMV infections were treated until two consecutives samples were below the level of Ag detection. If rejection therapies were initiated, low doses of ACV or VGCV were administered for 6 months as prophylaxis, which was done in the same manner as in the early phase.

CMV infection was defined as the presence of CMV-Ag, regardless of symptomatology, and CMV disease was diagnosed if CMV infection was present and accompanied by clinical signs and symptoms (Razonable and Humar, 2019). Furthermore, we compared the clinical characteristics between the patients who had CMV-Ag in the chronic phase and those who did not have it.

### 2.3 Immunosuppression therapy

We used the immunosuppressive triple therapy consisting of a calcineurin inhibitor as the base, a steroid and mycophenolate mofetil (MMF), as well as basiliximab for induction therapy. Everolimus (EVR) was used instead of steroid or MMF for some patients independently of the CMV-Ab status. A desensitization therapy consisting of antibody removal and rituximab treatment, in addition to the basic triple immunosuppression, was performed prior to ABO-incompatible KTx (Tasaki et al., 2009). For maintenance immunosuppressants, the trough levels of tacrolimus, cyclosporine A and EVR were adjusted to 3–5 ng/mL, 60–130 ng/mL, and 3–8 ng/mL, respectively. The doses of MMF and methylprednisolone were 500–1,000 mg/day and 4 mg/day, respectively. When rejection was clinically suspicious or diagnosed by biopsy, we started some of the following therapies: steroid pulse, rabbit anti-thymoglobulin, rituximab, plasmapheresis, or bortezomib. The combination of these therapies was decided according to the type of rejection and severity.

### 2.4 Statistical analysis

Comparisons between two groups were performed using the standard Student’s *t*-tests or Welch’s tests for continuous variables, and the Fisher’s exact tests for categorial variables. The multivariate analysis was performed using a multiple logistic regression model. Effects were considered significant if *P* < 0.05. All statistical analyses were performed using EZR (Kanda, 2013).

## 3 Results

### 3.1 Patients’ characteristics

The patients’ characteristics are shown in **Table 1**. We compared patients who experienced CMV-Ag and those who never had CMV-Ag during the chronic phase. The median duration of the follow-up after KTx was 80.6 (13.1–172.1) months. There was no significant difference in sex, age, type of donor (living or deceased), ABO-compatibility, and preemptive KTx between the two groups. EVR was used more often in patients that did not have CMV-Ag than in those that experienced CMV-Ag, although the difference was not statistically significant. CMV serostatus before KTx was not significantly different between the two groups. The rate of CMV infection within 1 year after KTx was significantly higher in patients that experienced CMV-Ag in the chronic phase than in those that did not have it (75.8% vs. 60.6%, *P* = 0.039). Thirty-nine recipients were CMV seronegative before KTx, and 22 out of 39 patients acquired CMV IgG Ab within 1 year after KTx. The fractions of the CMV seropositive patients at 1 year post KTx (the start of this study period) were not significantly different between patients with and without CMV-Ag. Asymptomatic CMV infection and CMV disease were observed 30.7% and 2.9% of all enrolled recipients in the chronic phase, respectively. Almost all (91.3%) patients with CMV-Ag had asymptomatic infection. The number of biopsy-proven rejections (BPR) in the early phase (within 1 year after KTx) was not significantly different between the two groups. However, BPRs in the chronic phase were frequent in patients that had CMV-Ag (*P* = 0.008). Furthermore, the incidence of graft function loss was significantly higher in patients who had history of CMV-Ag in the chronic phase than in those who did not present with CMV-Ag.

**Table 1:** Comparison of the clinical characteristics between the patients with or without positive CMV-Ag in chronic phase; univariate analysis.

	Total		CMV-Ag in chronic phase				p-value
	(n=205)		positive (n=69)		negative (n=136)		
Sex, n (%)							0.644
Male	135	(65.9)	47	(68.1)	88	(64.7)	
Female	70	(34.1)	22	(31.9)	48	(35.3)	
Age at KTx, median (range)	41.0	(3-72)	40.0	(3-72)	41.0	(4-68)	0.945
Follow-up period (months), median (range)	80.6	(13.1-172.1)	88.3	(13.9-169.3)	74.3	(13.1-172.1)	0.228
Annual number of CMV monitoring per patient, median	6.5		7.5		6.3		0.010
Cause of ESRD, n (%)							N/A
IgA nephropathy	47	(22.9)	24	(34.8)	23	(16.9)	
DM nephropathy	24	(11.7)	8	(11.6)	16	(11.8)	
CGN	11	(5.4)	5	(7.2)	6	(4.4)	
PCKD	10	(4.9)	2	(2.9)	8	(5.9)	
Reflux nephropathy	9	(4.4)	4	(5.8)	5	(3.7)	
FSGS	8	(3.9)	5	(7.2)	3	(2.2)	
Alport syndrome	8	(3.9)	2	(2.9)	6	(4.4)	
Renal hypoplasia	6	(2.9)	1	(1.4)	5	(3.7)	
MPGN	5	(2.4)	1	(1.4)	4	(2.9)	
Others	23	(11.2)	6	(8.7)	17	(12.5)	
Unknown	54	(26.3)	11	(15.9)	43	(31.6)	
Type of transplantation, n (%)							1.000
Living donor	162	(79.0)	55	(79.7)	107	(78.7)	
Deceased donor	43	(21.0)	14	(20.3)	29	(21.3)	
Age of donor, median (range)	55	(11-80)	55	(23-78)	55	(11-80)	0.205
Unrelated donor, n (%)	91	(44.4)	26	(37.7)	65	(47.8)	0.178
ABO-, n (%)							0.196
Compatible	161	(79.7)	58	(85.3)	103	(76.9)	
ncompatible	41	(20.3)	10	(14.7)	31	(23.1)	
Unknown	3		1		2		
PKT or non PKT, n (%)							0.100
PKT	57	(27.8)	14	(20.3)	43	(31.6)	
Non PKT	148	(72.2)	55	(79.7)	93	(68.4)	
Maintenance immunosuppression (%)							
FK	117	(57.1)	41	(59.4)	76	(55.9)	0.657
CYA	88	(42.9)	28	(40.6)	60	(44.1)	0.657
MMF	145	(71.1)	53	(76.8)	92	(68.1)	0.253
EVR	31	(15.2)	7	(10.1)	24	(17.8)	0.216
Other	9	(4.4)	3	(4.3)	6	(4.4)	1.000
Steroid	186	(91.2)	63	(91.3)	123	(91.1)	1.000
Anti-CMV IgG status before transplantation, n (%)							0.204
D+R+/D-R+	156	(76.1)	57	(82.6)	99	(72.8)	0.316
D+R-	29	(14.1)	8	(11.6)	21	(15.4)	
D-/R-	5	(2.4)	0		5	(3.7)	
Unknown (each of D or R)*	15	(7.3)	4	(5.8)	11	(8.1)	
CMV infection within 1y after KTx, n (%)	130	(65.7)	50	(75.8)	80	(60.6)	0.039
Anti-CMV IgG status at 1y post KTx, n (%)							
Positive	178	(86.8)	62	(89.9)	116	(85.3)	0.512
Negative	17	(8.3)	3	(4.3)	14	(10.3)	0.185
Unknown	10	(4.9)	4	(5.8)	6	(4.4)	0.735
CMV infection in chronic phase, n (%)							
Asymptomatic infection	63	(30.7)	63	(91.3)	0		N/A
CMV disease	6	(2.9)	6	(8.7)	0		N/A
Biopsy-proven rejection within 1y after KTx, n (%)							0.672
+	29	(14.1)	11	(15.9)	18	(13.2)	
-	176	(85.9)	58	(84.1)	118	(86.8)	
Biopsy-proven rejection in chronic phase, n (%)							0.008
+	26	(12.7)	15	(21.7)	11	(8.1)	
-	179	(87.3)	54	(78.3)	125	(91.9)	
Loss of graft function, n (%)							<0.001
+	13	(6.3)	11	(15.9)	2	(1.5)	
-	192	(93.7)	58	(84.1)	134	(98.5)	

KTx, kidney transplantation; ESRD, end stage renal disease; DM, diabetes mellitus; CGN, chronic glomerulonephritis; PCKD, polycystic kidney disease; FSGS, focal segmental glomerulosclerosis; MPGN, membranoproliferative glomerulonephritis; PKT, preemptive kidney transplantation; FK, Tacrolimus; CYA, Cyclosporin; MMF, Mycophenolate mofetil; EVR, Everolimus; D, donor; R, recipient.

*Five recipients were CMV seronegative before KTx.

### 3.2 CMV-Ag measurement

There were 6,345 measurements of CMV-Ag in 205 patients in the chronic phase. CMV-Ag monitoring continued over 10 years after KTx in half of the patients. Annual number of CMV monitoring per patient was 6.5 (median) (**Table 1**).

**Figure 1:**
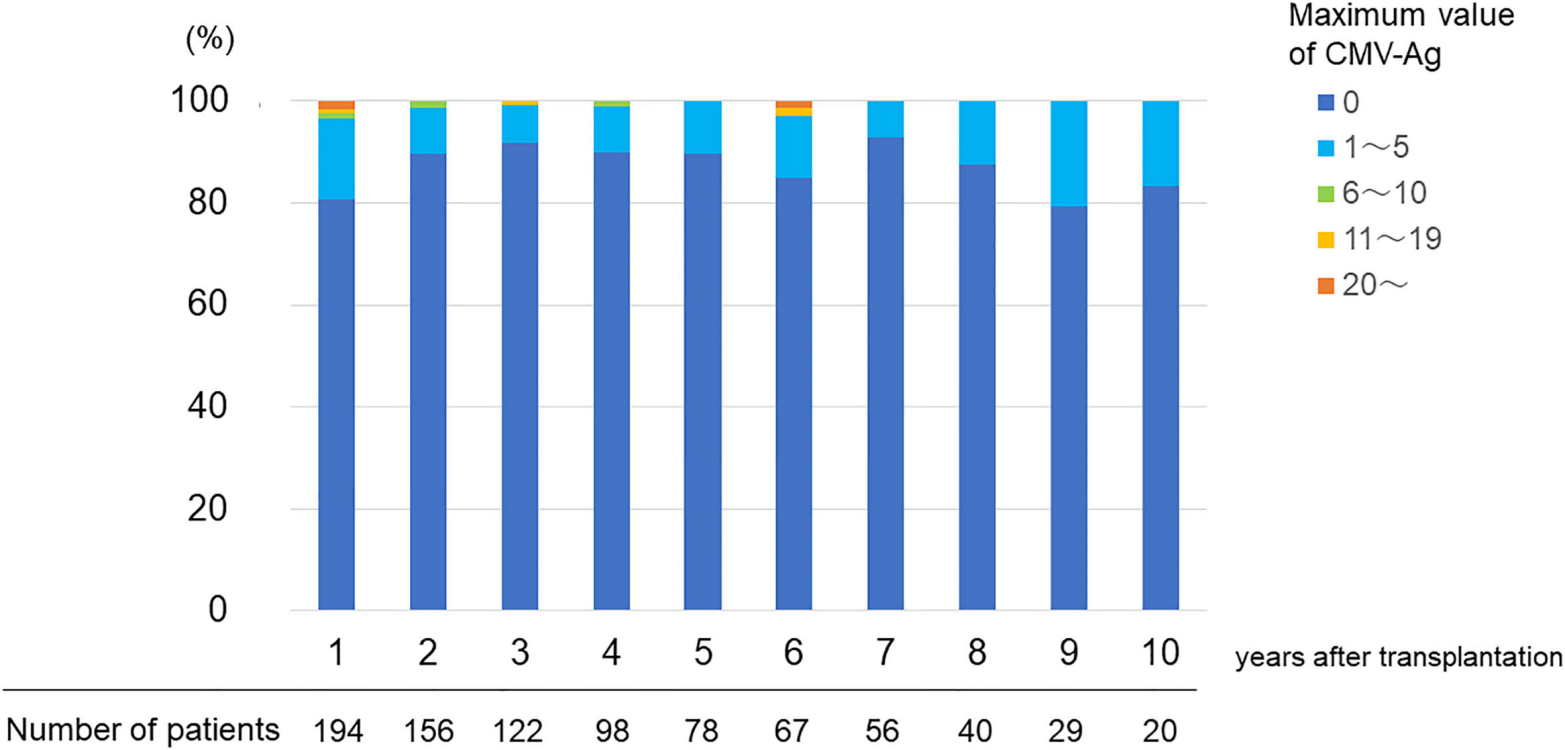
Maximum value of CMV-Ag in each year after transplantation.

Sixty-nine (33.6%) patients experienced CMV-Ag at least once in the chronic phase and 16 of them (23.2% of those who had CMV-Ag) often tested positive for CMV-Ag for several years. **Figure 1** shows the maximum value of CMV-Ag in each year after KTx. In most patients, the maximum values of CMV-Ag were zero. However, 10–20% of recipients experienced CMV viremia under the chronic immunosuppression. The rate of CMV viremia did not change even in the long-term phase after KTx. There were three patients who experienced the maximum value of CMV-Ag (> 10) at 1–2 years after KTx. The first patient was seronegative at the KTx, but the donor was seropositive (D+R−), so the patient experienced the first CMV infection 15 months after KTx (the maximum value of Ag was 95). The second patient experienced CMV-Ag immediately after the cessation of oral VCGV for the treatment of CMV infection that started less than 1 year after KTx, and VCGV was restarted (the maximum value of Ag was 24). The third patient was under the treatment for pneumonia due to pneumocystis jirovecii pneumonia (the maximum value of Ag was 15). These three patients did not develop chronic rejection episodes afterwards.

### 3.3 Treatment against CMV in chronic KTx recipients

**Figure 2:**
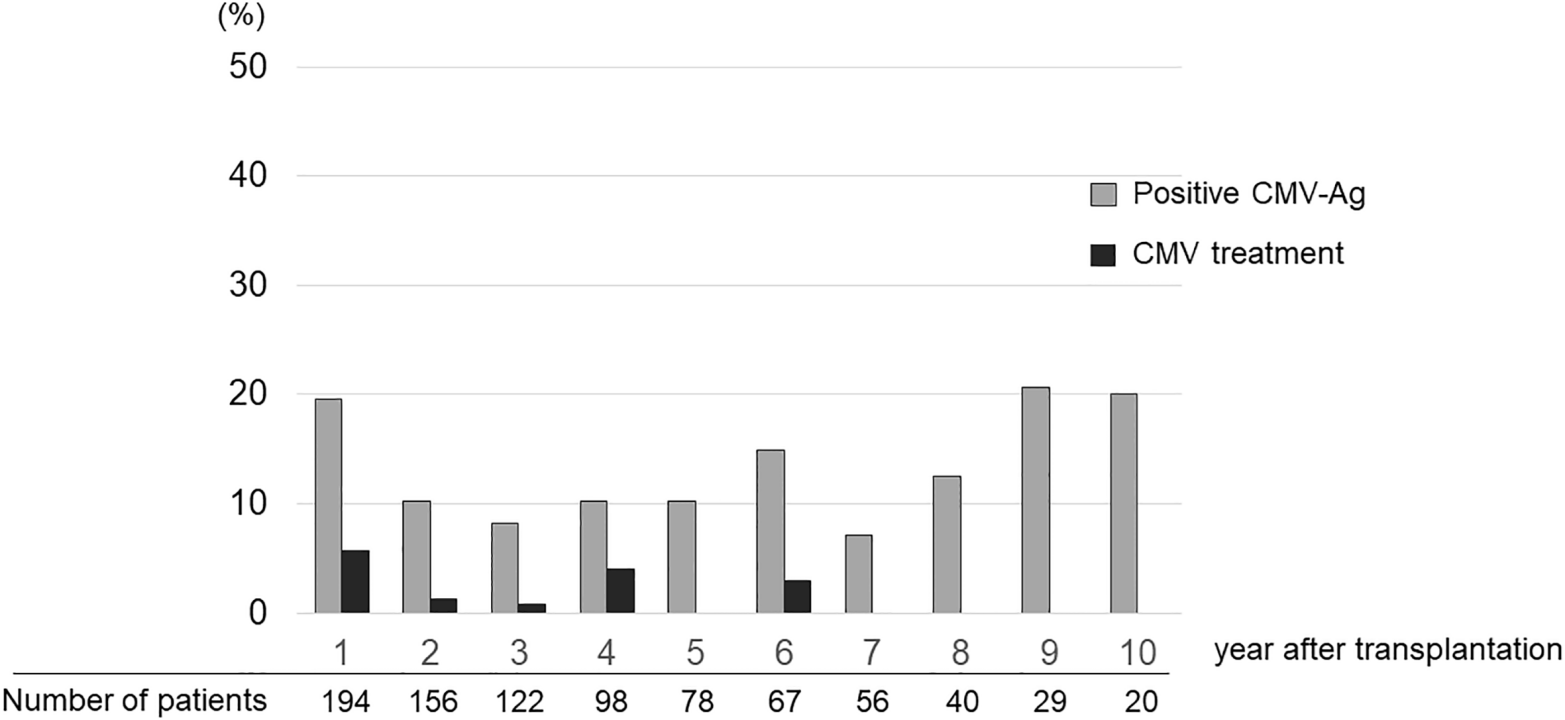
Ratio of patients who experienced CMV-Ag and were treated for CMV infection in each year after transplantation.

**Figure 3:**
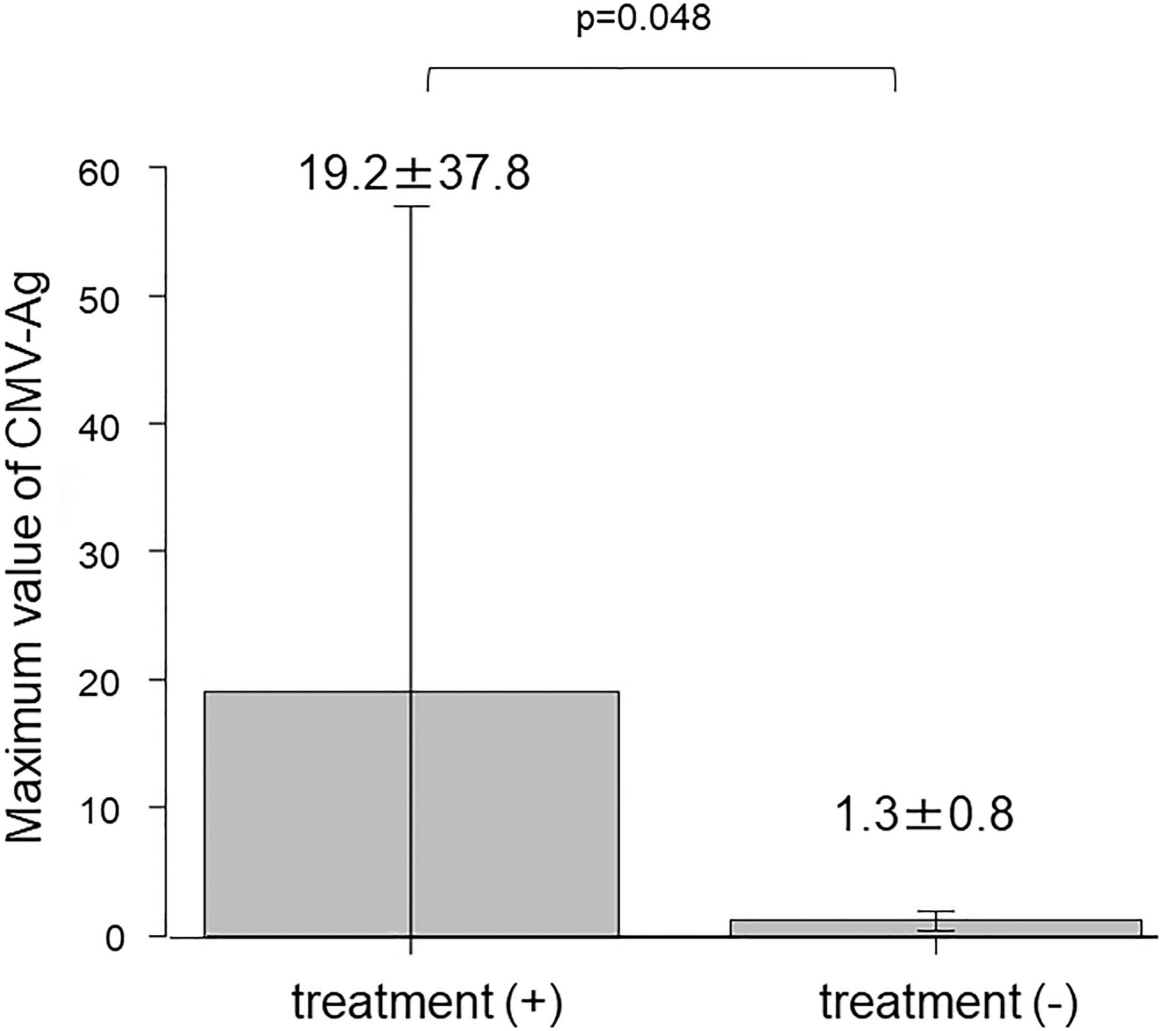
Comparison of the maximum value of CMV-Ag between treated and untreated patients.

The ratio of patients who received CMV treatment in each year was below 5% (**Figure 2**). A total of 20 treatment courses against CMV were performed in 19 cases. The median time after KTx when treatment against CMV was initiated was 23.1 months (range 12.9–144.7). Ten patients received treatment against CMV between 1 and 2 years after KTx, six — between 2 and 5 years, three — between 5 and 10 years, and one — after 10 years. There were six patients with CMV disease who presented with clinical signs that accompanied CMV-Ag. The symptoms included fever, cough, pneumonia, hematuria with the presence of decoy cells in urinalysis, elevation of serum creatinine level, and liver dysfunction. None of the participants in this cohort had gastro-intestinal CMV disease or CMV retinitis. The maximum value of CMV-Ag was significantly higher in the patients who underwent treatment against CMV than in those who did not receive such treatment (19.2 ± 37.8 vs. 1.3 ± 0.8, respectively, *P* = 0.048) (**Figure 3**).

### 3.4 Risk factors for CMV viremia in the chronic phase

We analyzed risk factors of CMV viremia during the chronic phase after KTx. **Table 2** presents univariate and multivariate analyses of factors associated with the incidence of CMV-Ag during this phase. The history of CMV infection in the early phase (within 1 year after KTx) and BPR during the chronic phase were found to be significant risk factors of CMV viremia. Because this study was started a year after KTx, the first factor was associated with ongoing CMV infection or reactivation within 1 year after KTx. Therefore, we focused on BPR as a risk factor of chronic CMV viremia.

**Table 2:** Univariate and multivariate analysis for positive CMV-Ag in chronic phase after KTx.

	Univariate analysis			Multivariate analysis		
	Odds ratio	[95%CI]	p-value	Odds ratio	[95%CI]	p-value
History of positive CMV-Ag in early phase (within 1y)	2.03	[1.05, 3.94]	0.036	2.32	[1.11, 4.83]	0.025
ABO-incompatible KTx	0.57	[0.26, 1.25]	0.163			
Donor age > 60 yrs	1.70	[0.92, 3.15]	0.091			
Recipient age <50 yrs	1.21	[0.46, 2.25]	0.554			
Deceased donor KTx	0.94	[0.46, 1.92]	0.864			
Unrelated donor KTx	0.64	[0.35, 1.15]	0.136			
PKT	0.55	[0.28, 1.10]	0.089			
CMV seropositive before KTx	2.23	[0.96, 5.18]	0.062	2.25	[0.76, 6.69]	0.145
CMV seropositive at 1y after KTx	2.49	[0.69, 9.01]	0.163	0.98	[0.17, 5.52]	0.978
BPR within 1 y	1.24	[0.55, 2.80]	0.600	0.99	[0.38, 2.59]	0.979
BPR in chronic phase	3.16	[1.36, 7.32]	0.007	3.10	[1.24, 7.73]	0.015
EVR usage in maintenance	0.52	[0.21, 1.28]	0.156			

KTx, kidney transplantation; PKT, preemptive kidney transplantation; BPR, biopsy proven rejection; EVR, Everolimus.

### 3.5 CMV-Ag and BPR in the chronic phase

Univariate and multivariate analyses were performed to identify the risk factors of BPR during the chronic phase (**Table 3**). The history of CMV-Ag during the chronic phase was a significant risk factor of BPR (*P* = 0.007), suggesting that CMV viremia is associated with immunomodulation against renal grafts, and acceleration of chronic rejection after KTx.

**Table 3:** Risk factors for BPR in chronic phase; univariate and multivariate analysis.

	Univariate analysis			Multivariate analysis		
	Odds ratio	[95%CI]	p-value	Odds ratio	[95%CI]	p-value
History of positive CMV-Ag in chronic phase	3.16	[1.36, 7.32]	0.007	2.77	[1.15, 6.66]	0.023
ABO-incompatible KTx	0.68	[0.22, 2.10]	0.507			
Donor age > 60 yrs	2.48	[1.08, 5.73]	0.033	1.78	[0.72, 4.38]	0.210
Recipient age < 50 yrs	3.06	[1.01, 9.26]	0.048	0.35	[0.08, 1.56]	0.170
Living donor KTx	1.53	[0.50, 4.71]	0.456			
Unrelated donor KTx	0.36	[0.14, 0.94]	0.036	0.74	[0.22, 2.45]	0.625
PKT	1.44	[0.60, 3.46]	0.409			
HLA mismatch ≥ 3	0.99	[0.42, 2.30]	0.976			
HLA mismatch ≥ 4	0.77	[0.27, 2.16]	0.615			
BPR within 1 y	1.54	[0.53, 4.46]	0.429			
CMV infection within 1y after KTx	0.70	[0.29, 1.67]	0.422			

KTx, kidney transplantation; PKT, preemptive kidney transplantation; BPR, biopsy proven rejection.

### 3.6 CMV-Ag and loss of graft function in the chronic phase

Subsequently, we investigated whether chronic CMV viremia affected renal graft survival. Both univariate and multivariate analyses results showed that the history of CMV-Ag and BPR during the chronic phase were independent risk factors of graft loss (**Table 4**), suggesting that the incidence of CMV-Ag during this phase was closely related to BPR and graft loss after KTx.

**Table 4:** Risk factors for graft loss; univariate and multivariate analysis.

	Univariate analysis			Multivariate analysis		
	Odds ratio	[95%CI]	p-value	Odds ratio	[95%CI]	p-value
History of positive CMV-Ag in chronic phase	12.70	[2.73, 59.10]	0.001	9.24	[1.83, 46.70]	0.007
ABO-incompatible KTx	1.19	[0.31, 4.55]	0.797			
Donor age > 60 yrs	1.66	[0.53, 5.15]	0.381			
Recipient age < 50 yrs	1.71	[0.45, 6.41]	0.429			
Living-donor KTx	1.49	[0.32, 7.00]	0.611			
PKT	0.77	[0.20, 2.89]	0.695			
CMV infection within 1y after KTx	2.16	[0.45, 10.50]	0.338			
BPR within 1 y	1.92	[0.49, 7.42]	0.347			
BPR in chronic phase	23.20	[6.45, 83.20]	<0.001	17.60	[4.57, 67.50]	<0.001
Unrelated-donor KTx	0.40	[0.11, 1.53]	0.182			
HLA mismatch ≥ 3	0.70	[0.23, 2.18]	0.543			
HLA mismatch ≥ 4	0.59	[0.13, 2.75]	0.499			

KTx, kidney transplantation; PKT, preemptive kidney transplantation; BPR, biopsy pr.

### 3.7 Relationship between CMV-Ag and rejection therapy in the chronic phase

**Figure 4:**
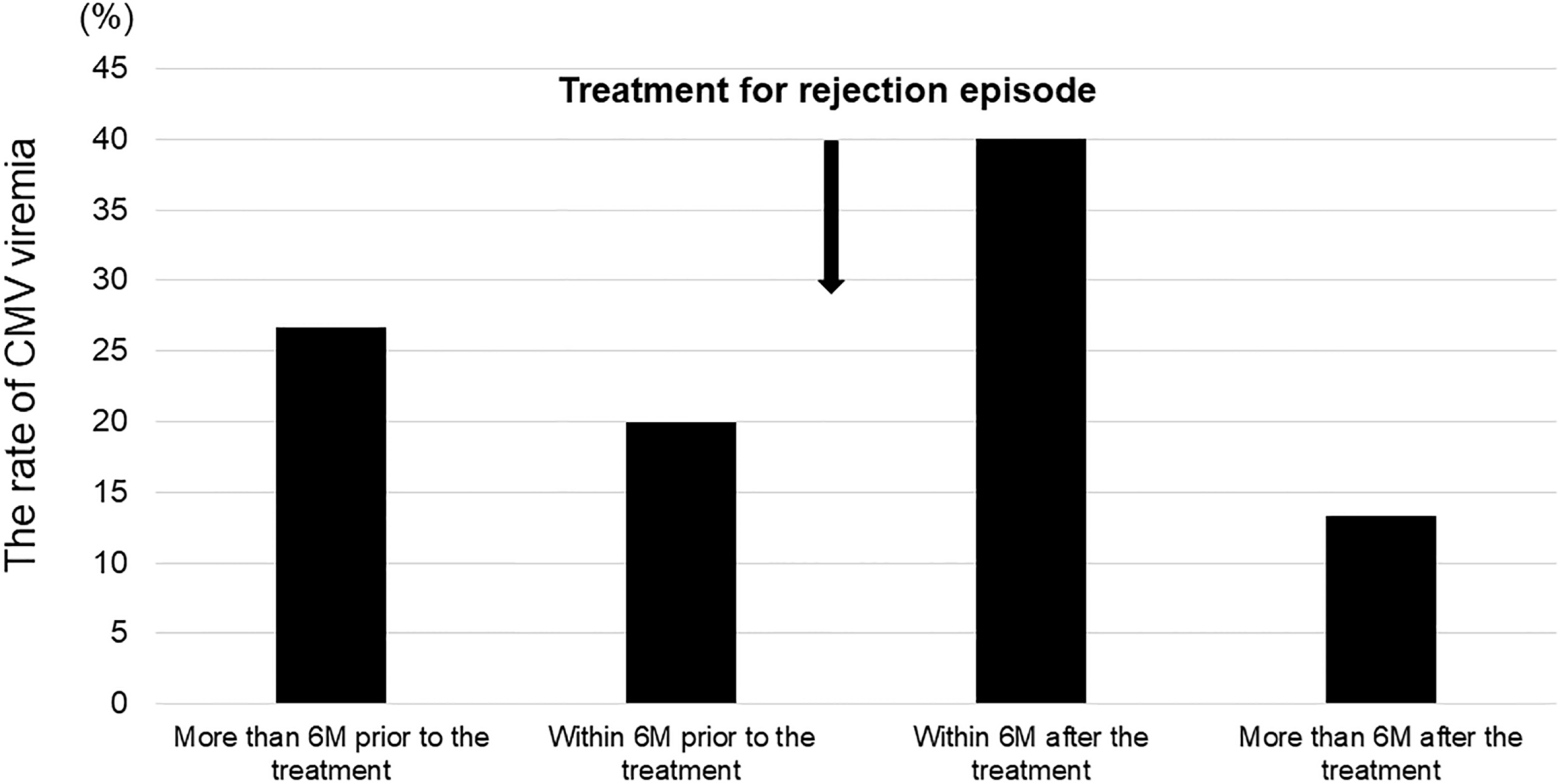
Timing of CMV infection in patients that received rejection therapies (n = 15).

There were 15 patients of positive CMV-Ag who received rejection therapy in the chronic phase. Twelve of these patients were diagnosed with chronic antibody mediated rejection. Of them, 40.0% experienced CMV-Ag within 6 months after rejection therapy, which was the most frequent occurrence (**Figure 4**). However, a total of 46.7% of patients had CMV viremia before rejection episodes in the chronic phase.

## 4 Discussion

The incidence of CMV infection after >1 year of transplantation in KTx recipients was analyzed in several reports. Browne et al. retrospectively reported on the incidence of late-onset CMV infection, defined as the first CMV infection diagnosed by various tests for CMV at ≥1 year after KTx (Browne et al., 2010). They combined all results monitored by different ways, such as the qualitative shell vial assay, antigenemia assay, and DNA PCR. Of the 2,489 recipients, 77 (3.1%) experienced the late-onset CMV infection. The mean time from KTx to late CMV infection was 54 ± 46 months. The majority of late CMV infection cases (70.1%) occurred between 1–5 years after KTx, whereas 19.5%, 5.2%, and 5.2% of cases were recorded in the periods of 6–10 years, 11–15 years, and >15 years following KTx (Browne et al., 2010). In another study, Viot et al. retrospectively analyzed CMV DNAemia using whole blood CMV-quantitative nucleic acid amplification test (QNAT) in 899 KTx recipients at least 2 years post transplantation (Viot et al., 2015). They reported CMV DNAemia incidence of 3.5% (32/899), including four symptomatic cases. They also reported the incidence of CMV disease over the year following their study (at least 3 years post transplantation) which was 0.9%. In our series, the frequencies of observing patients with CMV-Ag, CMV treatment, and CMV infection symptoms were 33.7% (69/205), 9.3% (19/205), and 2.9% (6/205) in the period ≥1 year post transplantation, respectively. The incidence of CMV infections in our study was much higher than that mentioned in the previous reports. However, the way of CMV infection monitoring, frequency of CMV testing, and duration of the follow-up were different in each study. In previous reports, CMV monitoring was performed when patients had signs of CMV infection in the chronic phase after KTx (Browne et al., 2010; Selvey et al., 2017; Rahimishahmirzadi et al., 2021), which were difficult to examine asymptomatic CMV infection. In the present study, most patients were monitored continuously (every 1–3 months) for CMV infection, which likely resulted in high incidence of detected CMV viremia (91.3% were asymptomatic) in the chronic phase. To the best of our knowledge, this is the first report to analyze in detail the impact of late CMV infection, including asymptomatic viremia, in patients after KTx. We found that 10–20% of patients receiving maintenance immunosuppression had CMV infections in each year post KTx, and this proportion was relatively stable over 10 years.

CMV IgG serology prior to KTx is an important factor of possible future CMV infection. CMV seronegative recipients who receive renal grafts from CMV seropositive donors (D+/R−) or CMV seropositive (R+) recipients have high risk of CMV infection, so antiviral prophylaxis therapy is recommended for such patients (Razonable and Humar, 2019). However, how CMV IgG serology prior to KTx affects the incidence of CMV infection in the chronic phase has not been properly elucidated. Nuansri et al. reported that the CMV D+/R− procedure has a significant risk for late-onset CMV infection in the recipient (Nuansri et al., 2021), but the number of CMV D+/R− pairs was only 2% in their cohort. In addition, the definition of late-onset CMV infection was later than 6 months after KTx in that study, which might have included cases that were considered early-onset CMV infection in our present study. It has been shown that even if the recipients have no anti-CMV IgG antibody before KTx, 50–60% of them develop CMV-specific immunity within 2 years after KTx (Humar et al., 2010; Halleck et al., 2017). In the present study, 82.1% (156/190) and 86.1% of the recipients were CMV seropositive before KTx and at 1 year post KTx (the start of this study), respectively. This is a common situation in the current era (Ishikawa et al., 2023). As we showed in the present study, CMV serostatus before KTx may not be a significant factor predisposing to CMV infection in the chronic phase. Our multivariate analysis showed that the history of CMV infection within 1 year after KTx and BPR in the period of >1 year post KTx were significant risk factors for CMV infection in the chronic phase. The former factor was related to ongoing CMV infection or reactivation from the early phase. Most of the cases with relatively high values of CMV-Ag (more than 6 positive cells per 150,000 leukocytes) were detected in 1–2 years after KTx (**Figure 1**). It was also previously demonstrated that rejection was a risk factor for subsequent development of CMV infection, even in the setting of antiviral prophylaxis, and this risk might persist for a year or longer after rejection treatment (Jorgenson et al., 2019). However, this relationship was observed only in the early phase after KTx. This situation was observed even in the chronic phase according to our results. In addition, 46.7% of our patients who suffered from chronic rejection had CMV viremia before the diagnosis of rejection, suggesting that CMV viremia might have accelerated chronic rejection. Early CMV infection was shown to significantly decrease long-term graft survival, however, long-term CMV monitoring was not performed in that study (Sagedal et al., 2004). Our data suggest that latent CMV reactivation might be associated with their chronic rejection. Immunomodulatory effect of CMV, such as activation of inflammation (Freeman, 2009) and innate immunity (Sen et al., 2020; Soleimanian et al., 2022), can contribute to graft rejection. CMV infection leads to an increase in natural killer (NK) cell-mediated reactivity toward allogeneic target cells and augments antibody-dependent reactivity, including anti-HLA antibodies (Michelo et al., 2017). In this study, 12 out of 15 patients (80%) with chronic CMV viremia and who received rejection therapy were diagnosed with chronic antibody mediated rejection. NK cells resulting from CMV viremia may primarily contribute to increased chronic rejection during the chronic phase after KTx. It has been shown that long-term changes in gene expression levels in peripheral blood cells after CMV infection have a prolonged impact on kidney allograft (Ahn et al., 2021). Anti-CMV T cell immunity levels substantially drop in KTx patients shortly after transplantation and in most individuals, pretransplant levels are never reached, resulting in the increased frequency of CMV reactivation (Abate et al., 2010; Abate et al., 2012). Preventing latent CMV infection may contribute to decreasing chronic rejection and graft loss.

This study has some limitations. First, this was a retrospective, single-center study. Basically, we monitored CMV Ag for all patients every 1–3 months as described in Materials and Methods. However, we examined CMV Ag more often in certain patients who had CMV infections and/or received rejection therapies as an exception. Second, we did not perform molecular assay, such as cytokines and immune cell properties. Further study will be necessary to establish the relationship between CMV infection and graft rejection in the chronic phase after KTx. Lastly, QNAT is recommended for detecting CMV infection in the current guideline (Razonable and Humar, 2019). The antigenemia assay was the only way that was covered by Japanese medical insurance during this study. However, the CMV pp65 antigenemia assay is comparable to CMV QNAT in its ability to provide rapid and sensitive diagnosis of CMV disease and guide treatment options (Caliendo et al., 2000; Pang et al., 2003; Razonable and Humar, 2019; Nakamura et al., 2022). The optimal cut-off value of CMV-Ag in the chronic phase after KTx is unclear. In this study, we used the lowest value (at least one positive cell per 150,000 leukocytes) obtained from the C10C11 CMV-Ag assay as the cut-off value (Boeckh and Boivin, 1998; Einollahi, 2012); however, further investigation is needed for the optimal cut-off value of CMV viremia during the chronic phase after KTx.

In conclusion, this is the first study to examine the incidence of CMV-Ag for 10 years post KTx. We showed that CMV-Ag was observed in 10–20% of chronic KTx recipients under the long-term immunosuppression. We demonstrated that latent CMV infections may be related to chronic rejection and graft loss after KTx. Long-term monitoring of latent CMV infections may help decreasing chronic rejection and graft loss. However, further studies will be necessary to determine what categories of recipients should be treated for latent CMV infection after KTx. There are also the issues of costs associated with long-term monitoring and prophylactic treatment. CMV vaccine will reduce latent CMV infection in the chronic phase and thereby decrease the incidence of chronic rejection (Streblow et al., 2015).

## Data availability statement

The raw data supporting the conclusions of this article will be made available by the authors, without undue reservation.

## Ethics statement

The studies involving human participants were reviewed and approved by the Ethics Committee of the Faculty of Medicine of the Niigata University (registration number: 2018-0287). Written informed consent to participate in this study was provided by the participants’ legal guardian/next of kin.

## Author contributions

SI, MT, KS, YN, MI, and KT managed patients, collected data and samples. SI analyzed data and performed statistical analysis. SI and MT prepared the manuscript. KT and YT revised the manuscript. All authors contributed to the article and approved the submitted version.
